# Risk perceptions of high-dose primaquine and tafenoquine among Plasmodium vivax malaria stakeholders in Ethiopia: a qualitative study

**DOI:** 10.1136/bmjgh-2025-021763

**Published:** 2026-06-29

**Authors:** Muthoni Mwaura, Nora Engel, Muhaba Kejela, Kansite Gellebo Korra, Tsegabehan Wodaj, Tamiru Shibiru Degaga, Benedikt Ley, Ric Price, Anja Krumeich, Kamala Thriemer

**Affiliations:** 1 Global and Tropical Health Division, Menzies School of Health Research, Darwin, Northern Territory, Australia; 2 Department of Health Ethics and Society, Care and Public Health Research Institute, Maastricht University, Maastricht, LI, Netherlands; 3 Athena Institute, Vrije Universiteit Amsterdam, Amsterdam, Limburg, The Netherlands; 4 Department of Sociology and Social Anthropology, Arba Minch University, Arba Minch, Ethiopia; 5 Department of English Language and Literature, Arba Minch University, Arba Minch, Ethiopia; 6 College of Medicine & Health Sciences, Arba Minch University, Arba Minch, Ethiopia; 7 Division of Education, Menzies School of Health Research, Darwin, Northern Territory, Australia; 8 Centre for Tropical Medicine and Global Health, Nuffield Department of Medicine, University of Oxford, Oxford, UK; 9 Mahidol Oxford Tropical Medicine Research Unit, Faculty of Tropical Medicine, Mahidol University, Bangkok, Thailand

**Keywords:** Malaria, Treatment, Global Health, Qualitative study

## Abstract

**Background:**

Adherence to radical cure for *Plasmodium vivax* remains a major challenge for malaria control programmes. Novel regimens, such as 7-day high-dose primaquine regimen and single-dose tafenoquine, may improve treatment adherence and antirelapse effectiveness but can increase the risk of haemolysis in individuals with glucose-6-phosphate dehydrogenase deficiency. Stakeholder (ie, policymakers, providers, patients, etc) perceptions of these risks may significantly influence the acceptability and uptake of these regimens. Understanding these perceptions is essential for policymakers to design effective communication and implementation strategies of novel radical cure strategies.

**Methods:**

Guided by qualitative methodology and a risk perception lens, this study explored *P. vivax* malaria stakeholders’ experiences and interpretations of novel radical cure regimens. Between February and September 2023, 58 semistructured interviews and 6 focus group discussions were conducted at Arba Minch General Hospital and three health centres and health posts within the Gamo Zone of the South Ethiopia Regional State: Lante, Shecha and Shele. Participants included clinical trial staff and participants, health centre personnel, health extension workers and routine care patients.

**Results:**

The 7-day-high-dose primaquine regimen and single-dose tafenoquine were viewed as promising solutions to the adherence challenges of the standard 14-day primaquine regimen; however, participants raised concerns about the effectiveness of the shortened treatment duration and the safety of the increased daily dose of primaquine. A risk perception lens revealed that concerns about effectiveness and safety were influenced by prior public health messaging emphasising completion of the full 14-day regimen alongside fears of overdosing and drug-induced haemolysis. Pill characteristics, including number, shape and colour as well as fears of overdosing and drug-induced haemolysis also contributed to apprehension about the safety of these regimens.

**Conclusion:**

Participants’ perceptions of shortened radical cure regimens were shaped by concerns about effectiveness and safety, influenced by prior treatment messaging and pill characteristics such as higher dosing and pill burden. A risk perception lens can inform implementation and communication strategies for novel regimens, where addressing user perceptions alongside practical improvements, such as reducing pill burden, is essential for optimising uptake and adherence.

**WHAT IS ALREADY KNOWN ON THIS TOPIC**

- The low total primaquine dose (3.5 mg/kg) for the radical cure of *Plasmodium vivax* malaria is associated with reduced antirelapse efficacy, and adherence to the full 14-day course is often poor when treatment is unsupervised, further limiting effectiveness.
- In response, the World Health Organization’s 2024 updated antimalarial guidelines include a 7-day-high-dose primaquine regimen and single-dose tafenoquine, both of which have the potential to improve adherence and relapse prevention but can increase the risk of haemolysis.

**WHAT THIS STUDY ADDS**

- Applies a risk perception lens to explore how key stakeholders perceive the potential risks of these novel *vivax* treatment regimens, highlighting concerns about the reduced treatment duration associated with reduced efficacy because of prior messaging about the importance of prolonged treatment.
- Additional concerns identified included apprehension about the increased pill burden for high-dose primaquine associated with ‘overdosing’.

**HOW THIS STUDY MIGHT AFFECT RESEARCH, PRACTICE OR POLICY**

- Highlights the importance of communication strategies that address perceptions of effectiveness and safety when introducing shortened radical cure regimens for vivax malaria.
- Suggests that implementation approaches should engage communities in building understanding around new treatment regimens and consider patient concerns related to dosing and pill burden.

## Background

 Malaria remains a leading cause of morbidity and mortality in low-income countries, and in 2023 accounted for an estimated global burden of 263 million cases and 597 000 deaths.^1^ Of the six *Plasmodium* species that infect humans, *Plasmodium vivax* is the most geographically widespread species, with an estimated 9.2 million *P. vivax* malaria cases occurring worldwide each year.^1^ In 2023, Ethiopia accounted for 23% of global *P. vivax* cases and 98% of cases of almost 1 million cases reported from Africa.^1^

*P. vivax* malaria presents a unique challenge to malaria control efforts, due to its ability to recur weeks or months after the initial infection.^2^ Relapse, triggered by the reactivation of dormant liver-stage hypnozoites, is the primary cause of recurrent parasitemia and is estimated to account for up to 80% of *P. vivax* recurrences.^3^ In addition to causing febrile illness, relapses contribute to the cumulative risk of severe anaemia, malnutrition and growth delays in children as well as miscarriages, premature delivery and stillbirth in pregnancy.^4 5^ Furthermore, relapses sustain ongoing malaria transmission, posing a significant barrier to malaria elimination.^6^

Effective treatment of *P. vivax* malaria involves targeting both blood-stage parasites and dormant liver-stage hypnozoites, a drug combination known as radical cure.^7^ The only drugs capable of clearing hypnozoites are the 8-aminoquinolines primaquine (PQ) and tafenoquine (TQ), both of which can cause severe haemolysis in individuals with glucose-6-phosphate dehydrogenase (G6PD) deficiency.^7^ An estimated 400 million people worldwide are G6PD deficient, with significant geographical overlap between areas with high prevalence and malaria-endemic regions.^8^

To reduce the risk of haemolytic complications, particularly in settings where G6PD testing is not widely available such as Ethiopia, a 14-day low-dose PQ regimen (3.5 mg/kg total dose) is used.^9^ However, the low total dose is associated with reduced antirelapse efficacy.^10 11^ Moreover, adherence to the full 14-day course is often poor when treatment is unsupervised, further limiting effectiveness.^10^

In response to these limitations, the WHO included 7-day high-dose PQ (1 mg/kg/day; 7 mg/kg total dose) and single-dose TQ (300 mg) in their 2024 updated antimalarial guidelines.^7^ These regimens offer the potential for improved adherence and relapse prevention, but they also potentially carry a higher risk of haemolysis in individuals with reduced G6PD activity, due to the higher daily doses of PQ and the extended half-life of TQ.^12 13^ Consequently, the current WHO guidelines recommend quantitative point-of-care testing to identify and exclude individuals with intermediate and severe G6PD deficiency defined as an enzyme activity less than 70%.^7^ Ethiopia has yet to implement these revisions, the main barrier is the limited availability and costs of G6PD testing. The national prevalence of G6PD deficiency is relatively low but varies considerably across the country, with the A-variant reaching almost 5% in some locations.^14 15^

Transitioning to shorter, higher-dose radical cure alternatives will require consideration of financial, epidemiological and health system factors. Even after adoption into policy, their actual use and uptake will be shaped by a complex interplay of factors, including how different stakeholders (ie, policymakers, providers, patients, etc) perceive these regimens and the risks they pose.^16^ Risk perception literature, an interdisciplinary and evolving field, offers a useful lens for understanding how individuals interpret and respond to novel treatment regimens. Initially rooted in psychology’s psychometric and cognitive paradigms of the 1970s—which explored differences between expert and lay perceptions of risk—the field has since expanded to recognise risk as a socially-constructed and multidimensional phenomenon.^17^ Judgements regarding the safety, acceptability and feasibility of novel interventions are posited to vary between and within stakeholder groups and are shaped by biomedical considerations and a broader interplay of individual, cognitive, affective, institutional and cultural factors.^18^ These judgements have been shown to influence not only how interventions are understood but also the choices individuals make and the actions they take in response to them.^18^,^22^ Hence, understanding how health workers, patients and researchers perceive the risks associated with shorter and/or higher dose radical cure options is critical to facilitating their effective implementation.

## Methods

### Study aim and design

This study aimed to explore how key stakeholders involved in *P. vivax* malaria control programmes in Ethiopia perceive and understand novel radical cure regimens such 7-day high-dose PQ at a total dose of 7 mg/kg and single-dose TQ (300 mg). Specifically, it examined how their perceptions of risk shaped the acceptability of implementing such regimens within decentralised health facilities of the Ethiopian health system. Using a pragmatic qualitative approach, the study focused on the practical implications of these risk perceptions for routine malaria care, drawing insights from the lived experiences, contextual knowledge and institutional perspectives of stakeholders. Pragmatic qualitative research is an approach that uses the most practical and appropriate methods available to address a given research question, particularly when the aim is to understand a phenomenon, process or the perspectives of those involved.^23^ It integrates both description and interpretation, enabling the presentation of participants’ facts, feelings and experiences alongside the researcher’s interpretation.^23^

### Study setting and context

**Figure 1:**
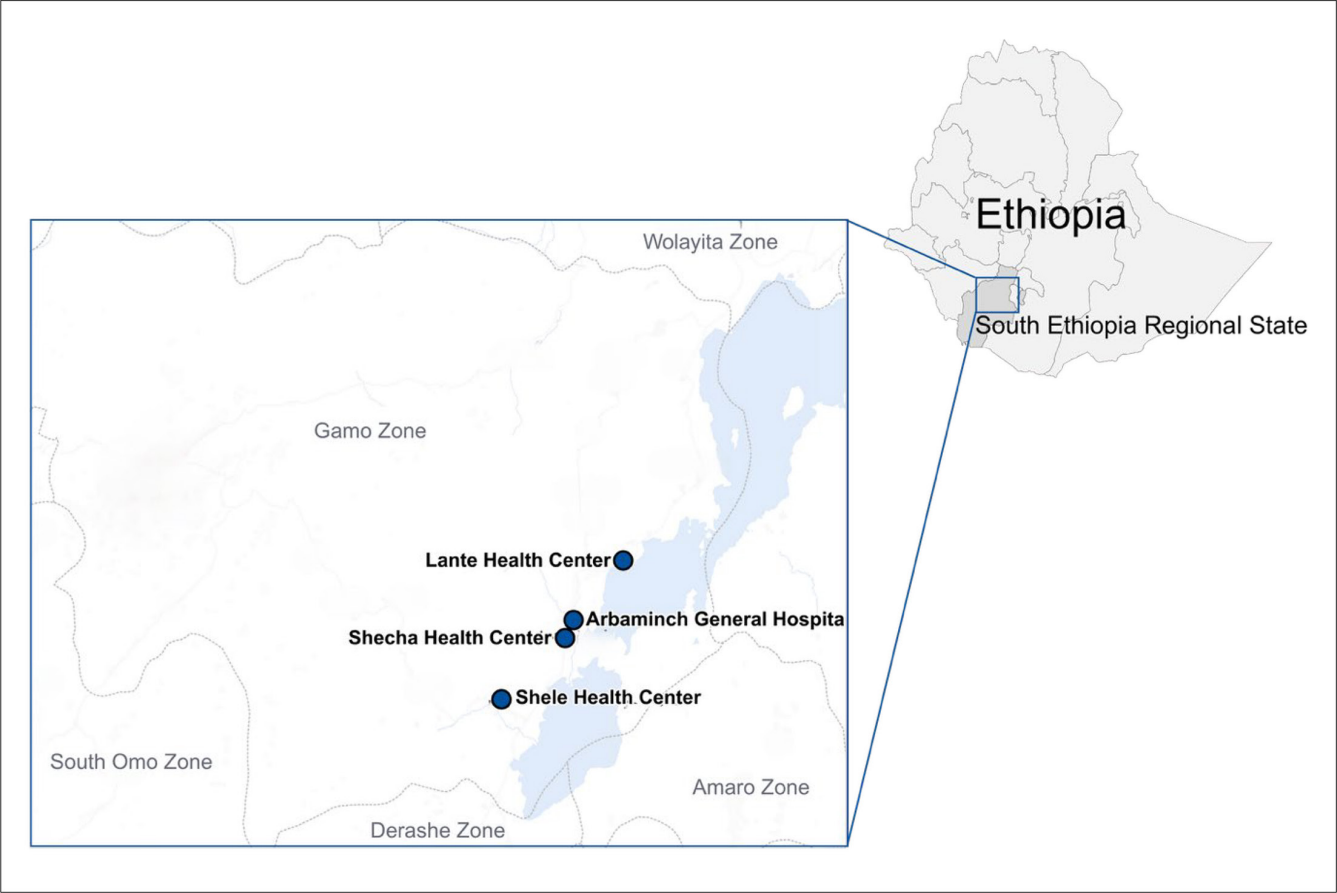
Map of study sites.

The study was embedded within the Ethiopian site of the EFFectiveness Of novel approaches to Radical cure with Tafenoquine and primaquine (EFFORT) clinical trial (NCT04411836), a multisite trial, which evaluated the effectiveness and safety of 7-day high-dose PQ and TQ.^24 25^ Study sites included in the qualitative study were the Arba Minch General Hospital and three health centres and associated health posts within the Gamo Zone: Lante, Shecha and Shele (figure 1). Gamo Zone is located within the South Ethiopia Regional State (formerly part of Southern Nations, Nationalities and Peoples Region), which has the highest documented pooled prevalence of *P. vivax* malaria*,* estimated at 10% (95% CI 8 to 46; 11% to 54%)^2^

In Ethiopia, health posts are the closest facilities to the community and serve as the first point of contact for basic health services.^25^ They are typically staffed by two female health extension workers (HEWs), who deliver a wide range of services related to family health, disease prevention and control, hygiene and environmental sanitation, health education and communication and non-communicable disease management.^26^ HEWs are expected to spend most of their time conducting household visits and engaging in community outreach. In rural areas, they normally have a lower secondary school education (grade 10) and undergo 12 months of training, whereas in urban settings, HEWs usually hold a clinical nursing diploma and receive an additional 3 months of training.^27^ Health centres, one level above health posts, provide outpatient care, including promoting, curative and rehabilitative services as well as inpatient care with a capacity of 10 beds for emergency and delivery services.^27^ These facilities are typically staffed by general medical practitioners (GPs), health officers, nurses (both diploma and Bachelor of Science (BSc) holders), midwives, pharmacy professionals and laboratory professionals^28^ (online supplemental file 1).

### Participants

Purposive and snowball sampling techniques were used to recruit participants involved in various aspects of *P. vivax* case management. Participants included EFFORT trial participants and staff, health centre facility staff involved in *P. vivax* case management (GPs, nurses, health officers, laboratory technicians), HEWs and patients who received routine *vivax* malaria care outside of the clinical trial. Individuals who were available at the time of recruitment were approached by MM and MK and invited to participate in the study. Of those invited, 76 individuals consented to participate, comprising 58 interviews and 6 focus group discussions (FGDs) (table 1).

**Table 1:** Study participants by stakeholder group

Stakeholder group	Total	Male	Female
EFFORT trial participants (*ep*)	22	16	6
Routine patients (*rp*)	7	5	2
EFFORT trial staff (*es*)	5	5	0
Health centre staff (*rs*)	35	29	6
Health extension workers (*hew*)	7	0	7
Total	76	55	21

EFFORT, EFFectiveness Of novel approaches to Radical cure with Tafenoquine and primaquine.

### Data collection

Individual interviews and FGDs were the main method of data collection and were used in a complementary manner to allow for a more comprehensive understanding of participant experiences and the social processes shaping them. Semistructured topic guides were developed using a risk perception conceptual model adapted by MM, drawing from Godovykh *et al*’s model of health risk perceptions in tourism and Siegrist and Árvais’s model of factors influencing risk perception^21 29^ (online supplemental file 2). This approach ensured a structured yet flexible exploration of participants’ perspectives on *P. vivax* case management, treatment adherence and risk perception.

Interviews and FGDs took place between February and September 2023. All interviews and FGDs were conducted by the research assistant (MK) in Amharic followed by debriefing sessions between MM and MK to reflect on emerging themes, adjust questioning techniques and ensure consistency across data collection. Data collection continued iteratively until no new insights or themes emerged from participant accounts, indicating data saturation. Interviews and FGDs were audio recorded and transcribed and translated (by MK, KGK and TW) and anonymised to ensure confidentiality.

### Data analysis

A thematic analysis approach was used to identify patterns and themes across the dataset, drawing on both inductive and deductive analytic approaches. Transcripts were first read for familiarisation, and initial codes were generated inductively to develop a codebook. Deductive codes informed by the risk perception model used to develop the topic guides were subsequently incorporated into the codebook. Coding was conducted using NVivo14. Codes were then reviewed, grouped into broader categories and refined into themes that captured patterns across the data set. These were used to iteratively generate outputs of code combinations in NVivo14 (ie, queries) to then inform the writing of memos (ie, a summary of ideas, insights, interpretations and understandings). Data presented are linked to participant type using unique codes structured as: participant type—unique identifier (interviews) and FGD—location (FGDs) (table 1).

### Reflexivity

Our multidisciplinary team combines local and international expertise in qualitative and malaria research. MM, a Kenyan qualitative researcher based in Nairobi, contributed regional cultural insight that supported adaptation of research tools to the local context despite not speaking the local languages used in the study. MK, KGK and TW, members of the study community, provided localised qualitative research experience, strengthened participant trust and enriched interpretation of findings. NE and AK, based in Netherlands, contributed extensive expertise in qualitative and ethnographic methodologies in global health. TD, the Ethiopia EFFORT clinical trial site lead, brought malaria research expertise, while BL, RP, and KT, based in Australia, provided senior guidance and global perspectives on vivax malaria research and dissemination. Online supplemental file 3 further outlines the team’s positionality in relation to this research.

### Patient and public involvement

In 2022, before commencing the study, the research team visited each prospective public health facility to introduce the study, outline its aims and discuss potential participation. These visits helped the team understand existing *P. vivax* case management practices and informed the study design, including adding questions to the topic guides based on issues raised during these discussions with healthcare professionals.

## Results

### Participant characteristics

A total of 76 participants were involved in the study, including 22 EFFORT trial patients (16 male, 6 female; 11 from TQ arm, 6 from 7-day high-dose PQ arm, 5 from 14-day low-dose PQ arm) and 7 routine *P. vivax* patients (5 male, 2 female). Health professionals included 5 EFFORT study staff (all male), 35 health centre staff (29 male, 6 female; 22 health officers, 9 BSc nurses, 1 GP, and 3 lab technicians) and 7 female HEWs. Overall, 72.4% (55/76) of the study participants were male, a pattern attributable to both the study sampling strategy and broader demographic and cultural trends in the health system. Males made up 56.3% (188/334) of the Ethiopian cohort of the EFFORT trial,^30^ hence their over-representation in this subsample may reflect greater availability or willingness of male patients to participate in interviews conducted by a male facilitator (MK). The gender distribution among health centre staff aligns with findings from a demographic survey in the neighbouring South Omo Zone, which reported 72.9% (229/314) of health centre workers were male.^31^ All HEWs in the study were female, consistent with Ethiopia’s national policy for the community health programme.

### Adherence challenges of 14-day PQ regimen

Participants across both FGD and in-depth interviews regarded 7-day high-dose PQ and single-dose TQ as promising solutions to the challenges of adherence associated with the current 14-day low-dose PQ regimen (RS-01, RS-04, RS-05, RS-10, RS-20). Reasons cited for poor adherence, either observed or experienced by participants, included discontinuation of medication on symptom resolution—particularly after initial therapeutic response following 3 days of schizonticidal treatment (EP-02, EP-07, EP-09, EP-10, EP-12-15, EP-17, ES-02, RP-01, RP-02, RP-04, RP-07, RS-02, RS-04, RS-20, RS-23); sharing of medication with others (EP-02, EP-13, ES-02); and saving medication for anticipated future illness (EP-09). Several participants also cited boredom or forgetfulness due to the prolonged duration of the treatment (EP-06, RP-05, RS-01, RS-02, RS-13, RS-16, RS-26, FGD-Lante, FGD-Shele, FGD-Shecha), fear of developing resistance to medication (EP-11) and stigma associated with taking medication for prolonged periods of time (EP-17). Additional factors included lack of awareness or insufficient counselling by health professionals on the importance of adherence (EP-07, EP-11, EP-12, EP-17, EP-18, RP-01, RS-10, RS-11, RS-28), adverse drug reactions such as gastric discomfort (RP-02, RP-03) and an aversion to the taste of the medicine (RP-05).

Although 7-day high-dose PQ and TQ were perceived as promising solutions to improve adherence, participants expressed concerns about both the shortened duration and increased dose of these treatments. Previous health messaging has emphasised the importance of completing a full 14-day course and this led many participants to question whether a shorter regimen could be more or even equally effective. Concerns were also raised on pill characteristics, fears of overdosing and understandings about the risks of haemolysis. The following sections explore these concerns in greater detail.

### Longer means better

When assessing the potential impact of shorter course radical cure regimens, participants frequently questioned the effectiveness of these regimens in eliminating the *P. vivax* hypnozoites. For some participants, the relationship between treatment duration and effectiveness appeared to influence the acceptability of shorter-course regimens (RS-07, RS-08, RS-13, RS-21, RS-23). When asked about changing the PQ regimen from the current 14 days to 7 days, one health officer at a health facility noted:

*The reason of giving the primaquine for fourteen days is to eradicate the hypnozoites which is found in the liver, and it is very good to take the medicine on long period of duration*. RS-10

A nurse echoed this view, reflecting a common perception that shortening the treatment course may be less effective because the medicine’s action depends on its half-life and sustained presence in the body:

*We have to see medicine until the end of the dose because it may continue treating the patient in the mean time of the process. A person could not get treated only by shortening the time and decreasing the dose of the medicine, but it matters the proper use of the medicine*. RS-18

Similarly, when asked if radical cure can be administered as a single dose in the form of TQ, a health officer responded:

*I don’t think it could prevent the relapsing incident; the reason of making it fourteen days is not to relapse and to go away completely from the liver, but the single dose can’t kill it entirely; thus, he has to take it for fourteen days*. RS-09

At the time of the study, most participants were unfamiliar with single-dose TQ, as it had not yet been included in Ethiopia’s treatment guidelines. When asked about the potential impact of a new single-dose radical cure treatment, some participants assumed it would be similar to the single-dose PQ dose currently provided for *P. falciparum* gametocytocidal treatment and therefore questioned its effectiveness for *P. vivax* (RS-01, RS-03, RS-18, RS-19). As a health officer noted, *I think it [single-dose treatment] cannot be able to eliminate the disease. It can be possible for falciparum to have single dose but it can’t be perfect for vivax* (RS-03). In a country endemic for both *P. falciparum and P. vivax,* there is an awareness that different treatments were required for each species. Because single-dose PQ regimen is already used for *P. falciparum*, this influenced how a similar strategy was perceived when proposed for *P. vivax*, raising concerns that it may not be ‘strong or long’ enough to fully clear the liver-stage parasites responsible for relapse.

Treatment duration was also understood to influence drug safety. Some participants perceived longer courses as not only more effective but also gentler on the body. As a patient explained using a metaphor of alcohol consumption:

*I think the medicine could work effectively if it is taken slowly through taking some time…It could have certain harm if we take it fast; a person who drinks more bottles of beer may stagger quickly than the one who takes a single bottle of beer; so, it could be effective if it is taken for a length of time*. RP-03

Similarly, duration’s relationship to safety was also framed in terms of drug half-life and the ability to stop treatment in case of an adverse reaction. A health officer expressed concern that a single dose might leave no opportunity to intervene before harm occurs:

*By taking single dose of the fourteen days medicine, it could eradicate it, but all the side effects we mentioned could be resulted at a time; so, the patient will not have the chance to interrupt it and some part of his organ may be damaged before coming to us. At a normal hemoglobin level, the patient could be anemic after he takes the medicine at a time, but if he takes the medicine gradually, we can stop it. Therefore, the long period of duration is better one*. RS-10

Indeed, because of the longer half-life of TQ, administration of the single-dose TQ regimen and high-dose PQ is only recommended for G6PD normal patients with activity >70%, requiring quantitative testing to guide drug administration.

### Too many pills

The effectiveness of radical cure depends on the total dose of PQ administered^11^; hence shortening the regimen to improve adherence requires a higher daily dose. In Ethiopia, the current regimen is 14-day low-dose PQ (0.25 mg/kg/day; total 3.5 mg/kg). The 7-day high-dose PQ regimen (1 mg/kg/day, total 7 mg/kg) therefore requires four times the daily dose to halve the duration. Perceptions of the safety of 7-day high-dose PQ were shaped by understandings of treatment dosage, particularly the belief that higher doses taken over a shorter period could pose greater risks to the body. Participants’ concerns were shaped by how they made sense of issues such as overdosing and haemolysis as well as the physical characteristics of the pills themselves.

Most of the health professionals were aware of the potential side effects associated with PQ, particularly the risk of haemolysis (ES-02, RS-01-11, RS-13, RS-14, RS-16-20, RS-23, RS-25, RS-26). As one health officer noted:

*I know it [PQ] is very risky;. That’s the reason why the patient’s hemoglobin has to be checked, and urine color has to be tested. The worst thing is the anaemic person is more hemolyzed, and the patient can be more at risk taking medicine than the malaria infection itself; therefore, it is better to stop giving the medicine*. RS-05

For many health professionals, a higher daily dose than the ‘standard’ was understood to be accompanied by a greater likelihood of haemolysis and other drug-induced side effects such as gastric pain, highlighting a caution towards shorter, higher dose regimens (RS-2-04, RS-06-11, RS-16, RS-17, RS-19, RS-26, RS-22, R-23, RS-26, FGD-HEW). A health officer notes: *‘I think it could harm the patient because medicine can harm if you take out of the standard; rather than cutting down, it would be better to extend to minimize the side effects*’ (RS-22). Similarly, a nurse simply stated, ‘*Ok if we make it from 14 to 7 days, there can be the possibility of doubling the side effects*’ (RS-01). A health officer expanded on this concern, highlighting that if adverse reactions were to occur, it could undermine community trust in the health system:

*I think, it [7-day-high-dose] is not possible; imagine when a patient takes a double medicine the amount of dose that is supposed to take it in two days: no, this is an act of over dose… The client may also complain by the effect of the overdose and the society may get the information and question the credibility of the medicines. Ultimately, the society could reject the medical treatment assuming it as a poisonous medicine. There may be a revolving of false information about the medicine around the community*. RS-20

Interestingly, a distinction emerged between awareness of a potential harm and the actual experience with it. Many healthcare workers were aware of the potential risks associated with PQ, which frequently informed their concerns of the tolerability of shorter, higher dose regimens. However, most reported that actual incidents of severe adverse events were rare or absent in their clinical experience (RS-01, RS-03, RS-04, RS-08, RS-09, RS-13, RS-14, RS-19, RS-21, RS-22, RS-24, RS-25). As a health officer explains:

*Yes, I have the idea; we have learned that patients with such deficiency should not be given the medicine because it may cause gum bleeding and stool colored. That is only in theory, but I have not practically seen its effect and process*. FGD-Lante

Though the contrast between perceived risk and observed hazard often did not dispel concerns about high dose regimens for most healthcare workers, for some it appeared to temper their apprehension. As one health officer reflected:

*I think it would be even better to reduce to 3 days than 7 days…because patients will not be bored and forget the drug… …if there is a research finding which recommends to reduce the days from 14 to 3 it would be nice….but it should be based on research finding…it is not about haemolysis…we did not see any significant effect…so the guideline can be changed in order to double the dose and reduce the number of days to 3 only*. FGD-Shele

Nonetheless, concerns about the tolerability of these treatments were experienced by some of the patients treated with 7-day high-dose PQ or TQ in the clinical trial, who reported gastrointestinal symptoms (EP-02, EP-05, EP-06, EP-12, EP-13, FGD-EFFORT Patient) and headaches (EP-02, EP-12). As two patients in the TQ arm expressed:

*The medicine is somewhat strong for me. There was some sort of feeling like tiredness or fatigue, and it burned me inside after taking the medicine*. EP-05

Conversely, others on 7-day high-dose PQ and TQ regimens reported generally positive experiences (EP-01, EP-07-10, EP-18), and particularly an appreciation for the rapid relief from symptoms (EP-01; EP-02; EP-10-12). As two patients who received 7-day high-dose PQ shared:

*It [taking the medicine] was good. There was no feeling of sickness or pain after taking the medicine.* EP-07

*There was no sickness or discomfort after taking the…The color of my urine was approaching red before coming to this center, and I told the doctor about it before starting medication. When I started the medicine to be taken for 7 days, the color became normal just after two days*. EP-09

It is important to note that patients in the EFFORT clinical trial received their treatment with a light meal and a drink to improve tolerability. Additionally, they were tested for G6PD deficiency prior to treatment, to minimise the risk of haemolysis.

Perceptions of the shorter and/or higher dose radical cure regimens were strongly influenced by the physical characteristics of the pills namely their number, colour, size and shape. Many patients could generally recall the number of tablets they had taken in recent or distant past, with notable accuracy (verifiable among EFFORT patients), even when they could not remember the name or precise duration of the regimen (EP-02-04, EP-06, EP-08-14, EP-17, EP-18, RP-03, RP-05-07, RS-06). This strong recall reflects a broader sensitivity to pill burden, where the quantity of tablets taken becomes a key reference point for treatment experience.^32^ As such, the number of pills administered as part of the 7-day high-dose PQ regimen elicited strong reactions, with several participants expressing concern about taking multiple tablets at once (FGD-EFFORT Staff, FGD-EFFORT Patient, FGD-Shele). After being shown the number of pills that they would have to take at once for the 7-day high-dose regimen (8-12 pills when a 7.5 mg formulation was used), a patient simply stated, ‘*Such number of medicines could have further side effects*’ (RP-03). Another expanded:

*I have never taken eight tablets at one time, and I am very suspicious and questions the reason when I am told to take many tablets at a time; I usually ask other people to confirm the prescribed dose when I am told to take four tablets of malaria at one time, and I do it after confirming the right dose of the medicine*. RP-04

For some, the number of pills triggered an association to intentional self-harm. One EFFORT patient shared: ‘*I was also afraid to take the dose…I watched on the films that some people swallow overdose to commit suicide*’ (FGD-EFFORT Patient). Another recalled their mother’s reaction to their treatment dosage during the trial:

*My mother attempted to stop me from taking the drugs…later on, we have called my sister’s husband who is a medical expert and he informed that it is a new form of medicine…she has seen the three types of drugs on my hand and said are you going to kill yourself?… she knows that people swallow overdose to commit suicide*. FGD-EFFORT Patient.

A health worker expanded on this association between pill number and perception of harm in the community:

*…the community believes that medicine is not only a drug but it is also a poison…there some people who commit suicide by taking overdose…so, the community have a concern that taking many pills is a poison which may kill or cause abortion*. FGD-Shele

Having had experience with administering and monitoring 7-day high-dose PQ, an EFFORT staff member noted that while the new regimen may address adherence challenges, the current pill formulation (7.5 mg) used in Ethiopia influences the acceptability of the high dose regimen:

*Recently, drug preparation has decreased…previous it was just 15mg…but the current preparation is 7.5mg…so, [the pill] number would be larger when they take high dose…I think a person above 70kg is expected to take 12 pills…so, this causes discomfort for the patient…this could be one of the challenges*. FGD-EFFORT Staff

‪Similarly, pill size, shape, colour and taste triggered associations (EP-02, EP-03, EP-08-11, EP-14, EP-15, EP-17, EP-18, RP-01-05, RP-07). For some patients, PQ’s small size, round shape and brown colour resulted in associations with treatment to induce abortions (RP-04). These pill characteristics served as key heuristics for assessing treatment strength, safety and acceptability.

### Making sense of risk

Patients developed strategies to manage the risks and side effects associated with radical cure treatment, drawing on both personal experience, social networks and expert advice. Some patients reported taking time off work during the treatment period to rest and manage side effects (EP-10, EP-18, RP-01, RP-03, RP-04). Many reported taking the medicine with food to ease gastrointestinal symptoms (EP-05, EP-06, EP-13, EP-14, EP-18, RP-01, RP-03, RP-04, FGD-EFFORT Patient) or drinking sweet beverages to mask the bitterness of the tablets (RP-05, RP-07). Others requested that the schizonticidal medicine be administered via injection, believing it would bypass gastrointestinal discomfort (RP-01, RP-07). As a patient notes, ‘*there are some patients who request the medicine to be given in the form of injection when the tablet becomes unsafe for them which is very common in our community*’ RP-01.

When faced with the pill count of a higher dose PQ regimen during the clinical trial, an EFFORT patient recalled managing their anxiety by rationalising the difference between the new dosage and ‘normal’ dosage as only two extra pills:

*Most of the time, one or two drugs are prescribed for patients…for example 4 pills of Coartem are prescribed at a time… I frustrated because they gave me 6 pills at a time to swallow here… you swallow 4 pills of Coartem at a time…because of this I convinced myself that 6 pills at a time is greater only by 2 pills*… FGD-EFFORT Patient

Other participants, when unsure about dosage or duration, reported turning to friends or neighbours with health training for advice and reassurance (RP-03, RP-04, FGD-EFFORT Patient). A patient recounts an experience seeking and following a neighbour’s advice on dosage, easing side effects and completing treatment:

*Though my appetite was very low, I tried to take the medicine by taking variety of foods and juices ahead of taking the medicine to protect the further harm of my stomach until I finished the medicine; I regularly eat food right now after I follow the constructive advice which is given by the neighboring health expert. In the meantime, did you interrupt the medicine? It was my intention to interrupt in in the second day, but the expert strongly advised me to finish as the prescribed time and dose, and I did it*. RP-03

Seeking reassurance from experts was further reflected in how participants understood and valued research—as a means to uncover, address and ultimately prevent the perceived risks associated with shorter, higher dose treatments (EP-07, EP-09, EP-18, RP-01, RP-03, RP-05, RS-01, RS-02, RS-04, RS-07, RS-08, RS-10, RS-14, RS-16, RS-18 −21, RS-23, RS-25, RS-26, FGD-HEW, FGD-Lante, FGD-Shele, FGD-Shecha). For example, an EFFORT patient’s risk perception was informed by their trust in research and scientific expertise:

*You know that people are afraid to take new medicine because it may affect your nerve or may have something bad. However, I am not afraid to take the new medicine. We are here in Africa; the malaria is prevalent in the rift valley where we live; any health staff that cures us has not come. These people have scientists and they have many experts who make research. So I believe that I would get something good. I know that the new medicine would not have negative effects because it must have been carefully prepared*. EP-18

For those with concerns regarding the shorter and/or higher dose treatment regimens, research was seen to address fears stemming from uncertainties and pill characteristics, as illustrated by a health officer and patient:

*Though I have not seen the nature of the medicine, it could have a bitter taste; it may also have further side effects. …It may fix inside the throat of the patient if its size is bigger than the previous one because there are some people who do not drink enough water though I have not seen its actual size, but it should do decided according to the result of a research study either to double or not*. RP-05

Similarly, drawing on past experiences with health education efforts to build community awareness and acceptance of new treatment, a health officer recounts:

*Let me add some points; as to me, tafenoquine should encouraged to introduced in Ethiopia which could be very effective to treat malaria, but the health expert should strive to create an awareness about the single dose through various health education because our community, by its nature, resist and challenge to accept a new thing and object*. FGD-Lante

## Discussion

Novel short-course and/or high-dose radical cure regimens such as 7-day high-dose PQ and TQ are promising solutions to the well-documented adherence challenges of the current 14-day PQ regimen. The adherence challenges reported in this study included early treatment discontinuation following symptom resolution, forgetfulness, side effects and limited patient awareness; these observations are consistent with previous literature on PQ adherence.^33^,^36^ However, stakeholder perceptions of these novel regimens and the risks that they may pose can significantly influence their acceptability and eventual uptake. Participants in our study raised concerns about the effectiveness (ie, risk of relapse) and safety (ie, risk of adverse events) of 7-day high-dose PQ and TQ, findings that echo other qualitative research on stakeholder perceptions of these regimens,^37 38^ perceptions were largely shaped by prior health messaging about the duration of treatment and the physical characteristics of the pills themselves. Understanding these risk perceptions is, therefore, crucial for informing policy, health messaging and guiding the effective implementation of shorter-course radical cure strategies.

### Longer means better: the legacy of 14-day messaging

The association between treatment duration and drug effectiveness is deeply embedded in the history of PQ use for radical cure of *P. vivax* malaria as well as the global and national messaging surrounding it. Although PQ has been in use since the 1950s, WHO formally recommended the 14-day low-dose regimen (0.25 mg/kg/day) in its 1981 malaria treatment guidelines.^39^,^41^ For the next four decades, this remained the global standard, accompanied by widespread emphasis on the need for adherence to the full 14-day regimen to achieve effective radical cure and prevention of relapses. In Ethiopia, PQ was used for more than 20 years before being removed from national treatment guidelines in 1990 for undocumented reasons.^42^ It was reintroduced in 2018 as part of renewed efforts to strengthen *P. vivax* case management and implement radical cure.^43^ This long-standing global emphasis on the 14-day regimen—alongside Ethiopia’s own historical use and experience with it, contributed to the formation of mental models in which treatment length has become equated with drug effectiveness.

The importance of prior messaging in shaping risk perception can be understood through the Social Amplification of Risk Framework (SARF). Developed in 1988, SARF is an integrative risk perception theoretical framework that explains how psychological, social, cultural and political factors interact to amplify or attenuate public perceptions of risk and its manageability.^43^ Since its inception, it has been applied as an interdisciplinary tool for analysing risk communication and informing policy across diverse contexts, including infectious disease outbreaks, emerging technologies and environmental issues.^17 44 45^ Within this framework, trust is defined as the willingness to rely on the authorities or institutions tasked with decision-making and action in the management of technology, environmental issues, medicine and other domains or public health and safety. Trust is posited as a key mechanism for reducing complexity and enabling decision-making, particularly when individuals have limited knowledge about a hazard.^46^ In this context, information from trusted institutions, such as WHO and national malaria programmes plays a crucial role in shaping how novel treatments are perceived.^47^ Long-standing messaging from these institutions emphasising the importance of completing the prolonged treatment to achieve radical cure led to the perception that longer treatments are inherently more effective. As such, effective communication around novel shorter course regimens must acknowledge and directly address these uncertainties to support acceptance and uptake (table 2).^48^

**Table 2:** Risk communication activities informed by SARF^43^

Finding	Recommendation
Longer means better	Acknowledge that the previously recommended 14-day regimen was based on the best available evidence at the time, while more recent locally-generated evidence indicates improved effectiveness of shorter regimens
Too many pills	Train health care workers to administer treatment with food and highlight benefits of higher dose regimen (ie, can complete treatment sooner)
Too weak to work, too strong to trust	Use plain language to explain treatment rationale, highlighting shared goals with the 14-day regimen (ie, safe and effective relapse prevention)
Rare but alarming	Share peer examples of effective haemolysis management with available resources and clarify that short-course high-dose regimens are only to be administered to those with>70% G6PD activity
Making sense of risk	Leverage trusted voices (community health workers, peer educators) and foster ongoing two-way engagement

G6PD, glucose-6-phosphate dehydrogenase; SARF, Social Amplification of Risk Framework.

### Too many pills: how high-dose PQ regimens trigger fear

In Ethiopia, PQ treatment is administered using a 7.5 mg tablet formulation. Whereas the current low-dose regimen requires adults weighing over 50 kg to take two tablets daily for 14 days, administration of the 7-day high-dose PQ regimen increases the dose to at least eight tablets per day for adults over 60 kg, and up to 12 tablets for patients weighing more than 70 kg. The substantial increase in pill burden evoked mental imagery associated with self-harm and abortion, which in turn triggered negative emotions such as fear or dread. As such, to support the uptake of 7-day high-dose PQ, participants’ accounts suggest that use of 15 mg formulation and reformulating PQ into higher strength tablets (eg, 30 mg formulation) would reduce the number of pills taken at once and mitigate affective responses linked to high pill volume.

Pill characteristics served as key heuristics for participants in assessing the strength, safety and acceptability of 7-day high-dose PQ and TQ, echoing findings from other studies that demonstrated that drug colour, size and quantity influenced patients’ perceptions of efficacy and potency.^33 37 49 50^ Originating in cognitive psychology, heuristics have become a key construct in risk perception research. They refer to cognitive shortcuts or simplified mental strategies that individuals use to make decisions and judgments efficiently, often through a process of attribute substitution (ie, a complex attribute is unconsciously replaced by a simpler, more readily available one).^48^ One example is the affect heuristic, which highlights the role of emotion in shaping how people evaluate risk. From this perspective, individuals often assess the acceptability of a hazard based on how it makes them feel; positive emotions signal lower risk, while negative emotions, such as fear or dread, indicate higher risk.^48^ This experiential mode of thinking is believed to draw from an internal ‘affect pool’—a reservoir of feelings and associations linked to mental representations of hazards.^47^ In this context, mental representations related to self-harm and abortion elicited negative emotions, which led participants to associate the increased pill burden with higher risk, and consequently to view it as unacceptable.

### Too weak to work, too strong to trust: conflicting perceptions of risk?

Participants in this study expressed two seemingly contradictory concerns: that the shorter regimens are less effective at preventing relapse than the 14-day low-dose PQ, but that the also posed greater safety risks. These opposing perceptions echo findings from research on vaccine hesitancy, particularly among parents who reject childhood immunisations, and more recently, in public responses to the COVID-19 vaccines.^22 51^ In both studies, non-immunisers perceived vaccines as both ineffective at preventing disease and actively harmful to the body. These perceptions that a drug is both too weak to help but too strong to trust point to broader dynamics in how people interpret risk.

The psychometric paradigm, a foundational approach to studying risk perception, helps to make sense of this contradiction. It posits that the qualitative characteristics of hazards influence how acceptable those hazards are to individuals.^21^ The paradigm maps hazards within a two-dimensional space defined by two key factors: unknown risk (ie, novel, not well understood, or psychologically distant) and dread risk (ie, uncontrollable, involuntary, catastrophic or likely to result in fatal consequences).^21^ Cognitive psychology research has shown that these subjective hazard characteristics often outweigh objective indicators like morbidity or mortality when people judge whether an intervention is safe or effective.^52^

Participants’ doubts about the effectiveness of the new regimens are influenced by their unfamiliarity. Both 7-day high-dose PQ and TQ depart from the well-established 14-day regimen. This unfamiliarity triggered scepticism: if the regimens are new and their long-term outcomes not fully known, they may be ineffective.^51^ On the other hand, concerns about safety were closely tied to characteristics of dread, including the possibility of severe side effects, irreversible harm and uncontrollable outcomes.^21^ Even in the absence of direct experience or widespread harm, unfamiliarity heightened doubt about efficacy, while dread amplified fears about safety, together producing a coherent, affect-driven perception of the drugs as both ineffective and dangerous.

### Rare but alarming: how the unknown and unseen shape perceived risk

Although all the patients enrolled in the EFFORT study were tested for G6PD deficiency, most could not recall what the test was for and its relevance to their treatment. Similarly, none of the routine patients interviewed had heard of G6PD deficiency. Most healthcare workers were aware of PQ-induced haemolysis, however only one participant, a GP, demonstrated a deep understanding of the prevalence of the deficiency and its role in triggering haemolysis following PQ administration. This general unfamiliarity with G6PD deficiency and the infrequent experience of PQ-induced haemolysis, alongside a heightened fear of its occurrence, present important considerations for the implementation of 7-day high-dose PQ and TQ.

Within the psychometric paradigm, both G6PD deficiency and PQ-induced haemolysis can be classified as an ‘unknown risks’—hazards that are novel, poorly understood or lacking observable or immediate consequences.^47^ The low prevalence of G6PD deficiency in Ethiopia^14 15^ and limited experience with PQ-induced haemolysis paradoxically contribute to elevated perceived risk, as individuals often rely on the availability heuristics when direct knowledge or experience is limited.^51^ Public health research has shown that unfamiliar medical conditions can evoke vivid mental images of worst-case scenarios, amplifying their perceived severity.^51^ This is compounded by affect heuristics, whereby the mere association of PQ-induced haemolysis with potentially fatal outcomes can elicit dread, even among individuals who have never personally encountered haemolysis. Here, individuals tend to focus more on the severity of the feared outcome than on its actual likelihood, a phenomenon known as probability neglect.^53^ Effective communication must therefore consider these cognitive–emotional responses by acknowledging fears while contextualising the rarity of adverse outcomes to build informed, trust-based acceptance of G6PD testing and novel radical cure regimens.

### Making sense of risk: the role of experience, trust and social networks

Our findings highlight that individuals do not absorb health information passively but actively engage in risk management strategies rooted in their own experiences, social networks and levels of trust in institutions and healthcare providers. Participants often relied on trusted actors—whether local health workers and family members (for patients) or national and global policy and research institutions (for healthcare professionals)—to guide their judgments and decisions, especially in the face of uncertainty or limited personal knowledge. SARF posits that trust functions as a central mechanism in how people interpret and respond to risk messages. When information about a hazard is incomplete or complex, individuals often substitute this gap with interpersonal or institutional trust as a proxy for certainty.^17^

The SARF posits that risk communication efforts must address the social, cultural and institutional contexts in which information is received.^48^ Applied to 7-day high-dose PQ and TQ, risk communication efforts around these treatments must be grounded in trust, not as a one-time event but as an ongoing process of relationship-building, engagement and mutual dialogue. Effective strategies include establishing clearly structured communication systems, training credible messengers who can deliver information sensitively and accurately and fostering two-way, deliberative engagement that acknowledges and validates public concerns.^54^ Importantly, this approach departs from the traditional deficit model, which seeks to ‘correct’ lay understandings to align with expert perspectives and instead emphasises co-constructed understanding. These trust-based, participatory approaches drawn from SARF (table 2) are critical for supporting the successful implementation and acceptance of novel radical cure regimens.

### Limitations

This study has certain limitations. First, not all members of the research team were fluent in Amharic. To ensure consistency and depth in data collection, debriefing sessions followed each interview and FGD to review content, identify emerging themes and refine subsequent questioning. Second, the high volume and duration of audio recordings made back translation of transcripts not feasible. Finally, transcription was undertaken by three translators, which may have introduced variations in style and interpretations. To minimise this risk, each translator initially completed a small set of transcripts that were jointly reviewed and discussed to identify and resolve inconsistencies before proceeding with the remaining material.

## Conclusion

The judgements individuals make about novel interventions critically shape their decisions and behaviours in response to those interventions. In this study, participants interpreted shortened radical cure regimens through existing understandings of malaria treatment, particularly prior messaging emphasising completion of a full 14-day PQ course. Insights from the risk perception literature offer a valuable lens for understanding how perceptions of effectiveness and safety are formed and how communication strategies can support implementation and acceptance. Ultimately, such strategies should be grounded in trust-based, participatory approaches that prioritise the co-construction of understanding with stakeholders, rather than aiming to correct perceived misconceptions or misinformation. While this study provides insights into how communication around novel radical cure regimens can be designed once they are adopted into policy, further research is needed to understand how policymakers perceive these regimens within broader financial, epidemiological and health system contexts, and how such perceptions influence policy adoption and implementation.

## Supplementary material

10.1136/bmjgh-2025-021763 online supplemental file 1

10.1136/bmjgh-2025-021763 online supplemental file 2

## Data Availability

All data relevant to the study are included in the article or uploaded as supplementary information.
