# Supplementary material for: Risk perceptions of high-dose primaquine and tafenoquine among Plasmodium vivax malaria stakeholders in Ethiopia: a qualitative study
Source: BMJ Glob Health. 2026 Jun 29;11(6):e021763. doi: 10.1136/bmjgh-2025-021763 (PMC13331116; doi:10.1136/bmjgh-2025-021763)
Supplement: online supplemental file 1 [file bmjgh-11-6-s001.pdf]

BMJ Global Health Author Reflexivity Statement

Adapted from Morton, B., Vercueil, A., Masekela, R., Heinz, E., Reimer, L., Saleh, S., Kalinga, C., Seekles, M., Biccard, B., Chakaya, J., Abimbola, S., Obasi, A. and Oriyo, N. (2022), Consensus statement on measures to promote equitable authorship in the publication of research from international partnerships. Anaesthesia, 77: 264-276. https://doi.org/10.1111/anae.15597

**Study conceptualisation**	
1. How does this study address local research and policy priorities?	The study addresses priorities related to the implementation and acceptability of novel radical cure regimens for *Plasmodium vivax* malaria in Ethiopia. By exploring stakeholder perceptions of effectiveness, safety, and communication around treatment, the research aims to inform locally relevant malaria policy and implementation strategies on the same.
2. How were local researchers involved in study design?	Local researchers visited prospective study sites to introduce the study, discuss participation and assess existing case management practices. Insights from these visits informed the study design and adaptation of tools to the local context.
**Research management**	
3. How has funding been used to support the local research team(s)?	Funding supported the involvement of local qualitative researchers and community based team members in participant engagement, data collection and data curation.
**Data acquisition and analysis**	
4. How are research staff who conducted data collection acknowledged?	MK, KK, TW, who are members of the study communities and contributed to data collection and curation, are recognized as members of the research team and acknowledged through authorship and contributorship.
5. How have members of the research partnership been provided with access to study data?	Study data were shared and discussed collaboratively across the research partnership. Researchers involved in data collection, analysis, and interpretation had access to relevant study materials to support collaborative analysis and interpretation of findings.
6. How were data used to develop analytical skills within the partnership?	The collaborative analysis process enabled researchers from different settings and levels of experience to jointly interpret findings and exchange research expertise.
**Data interpretation**	
7. How have research partners collaborated in interpreting study data?	Data interpretation was collaborative and informed by complementary perspectives.

	Community-based researchers contributed contextual understanding and participant insights. Kenyan and Ethiopian researchers contributed regional and implementation perspectives, and international collaborators contributed methodological and global health expertise.
**Drafting and revising for intellectual content**	
8. How were research partners supported to develop writing skills?	Manuscript drafting and revision were conducted collaboratively across the partnership, allowing team members to contribute to interpretation, writing, and revisions. Senior qualitative and malaria researchers provided mentorship and feedback throughout the writing process.
9. How will research products be shared to address local needs?	The team is committed to formally disseminating findings back to vivax malaria stakeholders in Ethiopia to support local radical cure implementation and communication strategies. Dissemination of findings has already taken place with local policymakers and malaria researchers during a stakeholder meeting in November 2025.
**Authorship**	
10. How is the leadership, contribution and ownership of this work by LMIC researchers recognised within the authorship?	MM, a Kenyan qualitative researcher based in Nairobi, is the first author of the paper and played a central role in study conceptualisation, contextualisation, analysis, and manuscript development. MK, KK, TW, and TD are recognised through authorship and contributorship for their roles in participant engagement, contextual interpretation, and contributions to the study and manuscript.
11. How have early career researchers across the partnership been included within the authorship team?	Early career researchers MM and MK were actively involved in study activities including data collection, analysis, and interpretation.
12. How has gender balance been addressed within the authorship?	The authorship team includes researchers of different genders across collaborating institutions. Notably, the first two authors and the senior last author are women, reflecting gender balance within key leadership roles across the research partnership.
**Training**	
13. How has the project contributed to training of LMIC researchers?	MM underwent qualitative methodological training through Charles Darwin University and Maastricht University prior to the study and subsequently supported refresher training

	on data collection techniques for MK as well as guidance on data curation for KK and TW.
**Infrastructure**	
14. How has the project contributed to improvements in local infrastructure?	Although the project was not primarily infrastructure-focused, it strengthened local qualitative research collaboration and engagement with vivax malaria stakeholders, supporting future locally relevant research and dissemination activities.
**Governance**	
15. What safeguarding procedures were used to protect local study participants and researchers?	The study was conducted in accordance with relevant ethical approvals and research governance procedures. MK and TD, with contextual knowledge and community familiarity, supported culturally appropriate participant engagement and trust-building. Informed consent procedures were followed for all participants and data were handled in line with approved confidentiality and data management processes. Additionally, during each interview or focus group discussion, participants were reminded that participation was voluntary, that they could stop the discussion at any time, and that they did not have to answer any question they were uncomfortable with. Participants were also given opportunities to ask questions and seek clarification from the study team during and after the interviews and focus groups. Ongoing communication within the research team supported researcher wellbeing and oversight throughout data collection and analysis.
