# Supplementary material for: Risk perceptions of high-dose primaquine and tafenoquine among Plasmodium vivax malaria stakeholders in Ethiopia: a qualitative study
Source: BMJ Glob Health. 2026 Jun 29;11(6):e021763. doi: 10.1136/bmjgh-2025-021763 (PMC13331116; doi:10.1136/bmjgh-2025-021763)
Supplement: online supplemental file 2 [file bmjgh-11-6-s002.docx]

**Supplementary Information for**

# Risk perceptions of high dose primaquine and tafenoquine among *Plasmodium vivax* malaria stakeholders in Ethiopia – a qualitative study

[Supplementary File 1](#_Supplementary_File_1:): Standard Staffing Composition of Public Health Centers

[Supplementary File 2](#_Supplementary_File_2:): Example Semi-structured Interview Topic Guide

[Supplementary File 3](#_Supplementary_File_3:): Reflexivity Statement

[Supplementary File 4](#_Supplementary_File_4:): SRQR Reporting Checklist

# **Supplementary File 1:** Standard Staffing Composition of Public Health Centers^^[[1]](#footnote-1)^-^[[2]](#footnote-2)^^

| Healthcare professional | Description | Expected number at health center |
| --- | --- | --- |
| General medical practitioners | Complete 6-7 years of university training after upper secondary school (grade 12), including medical internships | 1 |
| Health officers | Undergo 4 years of university-level clinical training and internship after grade 12 | 2 |
| BSc Nurse | Complete 4 years of university-level training after grade 12, or may upgrade from diploma level | 2 |
| Diploma Nurse | Typically undergo 3 years of training after lower secondary school (grade 10) | 5 |
| Midwives | Diploma midwives: 3 years of training at college level  BSc Midwives: 3 years of university-level training, typically after upgrading from diploma | 3 |
| Pharmacy Technician | Three years of college-level training after grade 10 | 2 |
| Laboratory professionals | Technologist: 3 years of university-level training after grade 12  Technician: 3 years of college level training after grade 10 | 3 |

# **Supplementary File 2**: Example Semi-structure Interview Topic Guide

**Introduction**: We are interested in your experience as a staff member of this health facility. Your experiences are important to improve care and treatment**. There are no right or wrong answers.** Your identity will remain anonymous and confidential and anything you tell us will not affect your position. With your permission, we will record this discussion so that we can write it down and translate it to English. If at any point during the discussion you want to take a break or stop completely, you are free to do so.

**Ensure informed consent form is signed**

| **Question** | **Probes [No need to use if question already answered]** | **Information we are looking for** |
| --- | --- | --- |
| **Background**   1. What is your position? 2. What responsibilities does your position entail? 3. What does a normal workday look like for you? [daily routine] 4. How long have you been working at this facility? | - 1. Can you describe a normal workday in detail [If not already described] | Background information about staff member, their role in the facility, their experience as a health worker in the facility |
| **Individual experience and perception of malaria**   1. Is malaria common in this area? If so, what type of malaria (falciparum, vivax, mixed?) 2. Has malaria personally affected you? If so how? [probe if they or a family member has ever had malaria. What was that experience like for them] 3. How does malaria affect this community? 4. What has been your experience with malaria diagnosis and treatment? | - 1. How has vivax impacted you as an individual?   2. How does vivax impact the community? | Individual’s general experience, perception of malaria burden, severity, impact |
| **Patient pathway before facility**   1. Who, what, and where is typically the first point of contact for a patient experiencing malaria symptoms | - 1. Does malaria diagnosis and treatment take place at community level (i.e. health post? Is this common?   2. Which patients are referred from the health post to the facility?   3. Can falciparum diagnosis and treatment take place completely at health post? Why or why not   4. Can vivax diagnosis and treatment take place complete at health post? Why or why not? | Perception of community health program role and capacity in malaria case management |
| **Malaria diagnostic process at facility**   1. What first happens when a patient arrives at the facility? | - 1. What first happens when a patient arrives at the facility?   2. What takes place during the clinical assessment of the patient? [Detailed description of what forms are filled, what questions are asked for patient history, is a physical examination conducted?]   3. Who is responsible for the clinical assessment?   4. Can malaria treatment be provided based on this assessment alone? Why or why not? | Detailed description of initial moments of patient at the facility |
| 1. Is parasitological confirmation required prior to treatment? [if answered in 10d, ask “What is part of the parasitological confirmation process”]? | - 1. Which malaria tests (Light microscopy (thick/thin blood films), [multi-species] RDTs] are used at the facility? If all are available, which tests are used and when? Are any non-malaria tests also concurrently done at this stage?   2. When is the test done?   3. Where is the test done?   4. By whom?   5. What samples are used?   6. Who collects the samples?   7. How (if necessary) are samples stored?   8. Who interprets the results of the test, specifically microscopy? Are you able to differentiate between vivax and falciparum?   9. What is the turnaround time for malaria testing? | Malaria parasitological procedures, capacity, knowledge at the facility |
| **Malaria treatment process at facility**   1. If found positive for malaria what management decisions are taken? | - 1. Specify management decisions for vivax vs for falciparum vs mixed   2. What management decisions are taken if:      - 1. Patient is pregnant        2. Patient is infant < 6 months        3. Patient is breastfeeding infant < 6 months   3. Are any doses given at the health facility under supervision? What happens in cases of vomiting after first dose | Malaria case management protocol and practice at facility |
| **Perception of alternative vivax treatment regimens**   1. Have you heard of any shorter and/or stronger treatments for vivax? If no, proceed to question 14. If yes, ask which ones they have heard about, what they think about them, then proceed to probes. 2. What do you think of the possibility of shortening the current PQ regimen from 14 days to 7 days treatment? 3. What do you think of the possibility of having a new, single-dose treatment for vivax? | - 1. What would need to change in your current case management protocol if the regimen for Vivax treatment became 7 days instead of 14?   2. What would need to change if it was administered as a single dose in the form of a new drug called Tafenoquine?   3. What would need to change if a shorter, 7-day PQ regimen increased the total dosage from 3.5mg/kg to 7mg/kg Would supervision and monitoring of patient need to change? If so, how. If not, why not? | Knowledge, attitudes, perception, acceptability of a shorter, stronger vivax treatment regimens |
| **G6PD testing**   1. What have you been told about the G6PD enzyme? | - 1. Can you describe how this deficiency affects malaria treatment?   2. Have you ever encountered a patient with G6PD deficiency? Is it common in this area?   3. Have you ever encountered a patient with haemolysis due to malaria treatment? If so, how did you know and respond?   4. Do you test for G6PD deficiency in your facility? Why or why not?   5. Do you have any experience with G6PD testing? If so, which tests have you used? Were they useful?   6. In your opinion, is it important to test for G6PD deficiency before prescribing vivax medicine? Why or why not?   7. How would a requirement to test for G6PD deficiency impact your work?   8. What kind of information would you like a G6PD test to tell you? [e.g. an exact number of G6PD activity, or just normal/deficient result]   9. What kind of test characteristics would make a new test more acceptable/usable in your facility? [e.g. cost, number of user steps, turnaround time etc]   10. If G6PD testing was required before administering medication, at which step of the diagnosis and treatment algorithm would you place it?   11. If G6PD testing was required before administering medication, at which level of the health system of the would you place it? Health center or hospital? Why?   12. What do you expect will happen as a result of implementing G6PD testing? | Knowledge, experience and perception of G6PD deficiency, testing and haemolysis? |
| **Follow-up & Day 3 Visit**   1. To their knowledge, is there a national policy to follow-up with patients that are given radical sure treatment? [i.e. primaquine 0.25mg/kg daily x 14 days]? [If no, skip to 18] | - 1. If yes, how long has this policy been in place?   2. Please describe in detail what is to be done according to the policy?   3. Was there a training conducted regarding this guideline? | Knowledge of the guidelines |
| 1. Currently at there facility, is there any follow-up of vivax patients given radical cure treatment | - 1. If no follow-up is done, whether they think there should be?   2. If yes, please describe process in detail e.g. Are they requested to return to the facility? If so, how many days after starting treatment? Do they usually return? Are they reminded to do so?   3. Have they encountered a patient experiencing haemolysis symptoms during this follow-up? | Current follow-up practice at facility |
| 1. If the guidelines required that a vivax patient should return 3 days after initiating treatment… | - - 1. What tests would you include during this visit?     2. What questions would you ask the patient during this visit?     3. Overall, would this be useful/beneficial? Why or why not?     4. How would this impact your work? How might it affect the overall workflow of the facility?     5. Are there any challenges that the staff at the facility may face?     6. Are there any challenges that the patients may face?     7. Would such a visit improve patient adherence?     8. Would such a visit improve patient safety?     9. What should happen during such a visit? Who within the facility should be assigned to conduct such a visit? What kind of information is necessary to find out during this visit?     10. How might adverse events or early warning signs be detected during the visit?     11. What operational challenges might exist in implementing a day 3 Pv patient visit?     12. How might the health facility staff respond to conducting the medical exam?     13. What would make it easier for patients to take part in this day 3 visit?     14. What would make it easier for the health facility staff? [probe--reduce workload for other activities, need of more staff, assign activities differently to the different staff?] | Perception of a day-3 follow-up visit policy |
| **Community health role**   1. What role does the health post/HEWs currently play in malaria case management? | - 1. Has this role changed over time?   2. Do you think HEWs could play a different role in case management? [probe for examples]   3. Might a HEW be assigned to a vivax patient to call or visit the patient to check up on them after their treatment? | Perception on current and potential role of community health extension workers in malaria case management |
| 1. Do you have any questions/ concerns or things that you would like us to know? | | |
| 1. Any suggestions of who we should also interview? | | |

# **Supplementary File 3**: Reflexivity Statement

### BMJ Global Health Author Reflexivity Statement

Adapted from Morton, B., Vercueil, A., Masekela, R., Heinz, E., Reimer, L., Saleh, S., Kalinga, C., Seekles, M., Biccard, B., Chakaya, J., Abimbola, S., Obasi, A. and Oriyo, N. (2022), Consensus statement on measures to promote equitable authorship in the publication of research from international partnerships. Anaesthesia, 77: 264-276. <https://doi.org/10.1111/anae.15597>

| **Study conceptualisation** | |
| --- | --- |
| 1. How does this study address local research and policy priorities? | The study addresses priorities related to the implementation and acceptability of novel radical cure regimens for *Plasmodium* *vivax* malaria in Ethiopia. By exploring stakeholder perceptions of effectiveness, safety, and communication around treatment, the research aims to inform locally relevant malaria policy and implementation strategies on the same. |
| 1. How were local researchers involved in study design? | Local researchers visited prospective study sites to introduce the study, discuss participation and assess existing case management practices. Insights from these visits informed the study design and adaptation of tools to the local context. |
| **Research management** | |
| 1. How has funding been used to support the local research team(s)? | Funding supported the involvement of local qualitative researchers and community-based team members in participant engagement, data collection and data curation. |
| **Data acquisition and analysis** | |
| 1. How are research staff who conducted data collection acknowledged? | MK, KK, TW, who are members of the study communities and contributed to data collection and curation, are recognized as members of the research team and acknowledged through authorship and contributorship. |
| 1. How have members of the research partnership been provided with access to study data? | Study data were shared and discussed collaboratively across the research partnership. Researchers involved in data collection, analysis, and interpretation had access to relevant study materials to support collaborative analysis and interpretation of findings. |
| 1. How were data used to develop analytical skills within the partnership? | The collaborative analysis process enabled researchers from different settings and levels of experience to jointly interpret findings and exchange research expertise. |
| **Data interpretation** | |
| 1. How have research partners collaborated in interpreting study data? | Data interpretation was collaborative and informed by complementary perspectives. Community-based researchers contributed contextual understanding and participant insights. Kenyan and Ethiopian researchers contributed regional and implementation perspectives, and international collaborators contributed methodological and global health expertise. |
| **Drafting and revising for intellectual content** | |
| 1. How were research partners supported to develop writing skills? | Manuscript drafting and revision were conducted collaboratively across the partnership, allowing team members to contribute to interpretation, writing, and revisions. Senior qualitative and malaria researchers provided mentorship and feedback throughout the writing process. |
| 1. How will research products be shared to address local needs? | The team is committed to formally disseminating findings back to vivax malaria stakeholders in Ethiopia to support local radical cure implementation and communication strategies. Dissemination of findings has already taken place with local policymakers and malaria researchers during a stakeholder meeting in November 2025. |
| **Authorship** | |
| 1. How is the leadership, contribution and ownership of this work by LMIC researchers recognised within the authorship? | MM, a Kenyan qualitative researcher based in Nairobi, is the first author of the paper and played a central role in study conceptualisation, contextualisation, analysis, and manuscript development. MK, KK, TW, and TD are recognised through authorship and contributorship for their roles in participant engagement, contextual interpretation, and contributions to the study and manuscript. |
| 1. How have early career researchers across the partnership been included within the authorship team? | Early career researchers MM and MK were actively involved in study activities including data collection, analysis, and interpretation. |
| 1. How has gender balance been addressed within the authorship? | The authorship team includes researchers of different genders across collaborating institutions. Notably, the first two authors and the senior last author are women, reflecting gender balance within key leadership roles across the research partnership. |
| **Training** | |
| 1. How has the project contributed to training of LMIC researchers? | MM underwent qualitative methodological training through Charles Darwin University and Maastricht University prior to the study and subsequently supported refresher training on data collection techniques for MK as well as guidance on data curation for KK and TW. |
| **Infrastructure** | |
| 1. How has the project contributed to improvements in local infrastructure? | Although the project was not primarily infrastructure-focused, it strengthened local qualitative research collaboration and engagement with vivax malaria stakeholders, supporting future locally relevant research and dissemination activities. |
| **Governance** | |
| 1. What safeguarding procedures were used to protect local study participants and researchers? | The study was conducted in accordance with relevant ethical approvals and research governance procedures. MK and TD, with contextual knowledge and community familiarity, supported culturally appropriate participant engagement and trust-building. Informed consent procedures were followed for all participants and data were handled in line with approved confidentiality and data management processes. Additionally, during each interview or focus group discussion, participants were reminded that participation was voluntary, that they could stop the discussion at any time, and that they did not have to answer any question they were uncomfortable with. Participants were also given opportunities to ask questions and seek clarification from the study team during and after the interviews and focus groups. Ongoing communication within the research team supported researcher wellbeing and oversight throughout data collection and analysis. |

MM is a Kenyan qualitative researcher based in Nairobi. Despite not speaking the local languages used in this study, her familiarity with local cultures and customs gained from growing up in neighboring Kenya facilitated the adaptation of research tools to the local context.

MK, KK, and TW are members of the community where the research took place. They have localized experience in qualitative research and deep contextual knowledge that informed participant engagement, facilitated trust-building, and enriched the interpretation of findings.

NE and AK, based in the Netherlands, bring vast expertise in qualitative research methodologies, having conducted numerous ethnographic studies across diverse settings and topics in global health.

TD, the Ethiopian site lead of the EFFORT clinical trial, contributes both local and global expertise in malaria research.

BL, RP, and KT are senior advisors based in Australia who contribute extensive expertise in vivax malaria research and a global perspective on the application of the study’s findings.

Collectively, our team represents a wide range of education and professional backgrounds from East Africa and abroad. We share commitment to collaborative, locally relevant, and impactful research, with a pledge to formally disseminate the findings back to vivax malaria stakeholders in Ethiopia.

# **Supplementary File 4**: SRQR Reporting Checklist

The SRQR reporting checklist

For checking that qualitative health research articles can be understood and used by everyone

| 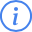 Note |
| --- |
| If you have not used a reporting guideline before, read about [how and why to use them](https://resources.equator-network.org/about/reporting-guidelines.html) and check whether SRQR is the [most applicable reporting guideline](https://resources.equator-network.org/reporting-guidelines/srqr/index.html?#applicability) for your work.  Reporting guidelines are most useful when used early in research. When writing a manuscript or application, consider using the [Full Guidance](https://resources.equator-network.org/reporting-guidelines/srqr/index.html) where you’ll see explanations and examples for each item.  After writing, demonstrate adherence by completing this checklist:   1. Specify where each item is described (see [Note 1](https://auc-word-edit.officeapps.live.com/we/wordeditorframe.aspx?ui=en-GB&rs=en-AU&wopisrc=https%3A%2F%2Fcharlesdarwinuni.sharepoint.com%2Fteams%2FClinicalTrials%2F_vti_bin%2Fwopi.ashx%2Ffiles%2Ff10e2025357e4157ab3983f16d92bb11&wdenableroaming=1&wdfr=1&mscc=1&hid=C4CF15A2-80A9-7000-82FB-75B1748C5A36.0&uih=sharepointcom&wdlcid=en-GB&jsapi=1&jsapiver=v2&corrid=59a17e2b-b656-7e7f-4a3d-3bebec4f37d9&usid=59a17e2b-b656-7e7f-4a3d-3bebec4f37d9&newsession=1&sftc=1&uihit=docaspx&muv=1&ats=PairwiseBroker&cac=1&sams=1&mtf=1&sfp=1&sdp=1&hch=1&hwfh=1&dchat=1&sc=%7B%22pmo%22%3A%22https%3A%2F%2Fcharlesdarwinuni.sharepoint.com%22%2C%22pmshare%22%3Atrue%7D&ctp=LeastProtected&rct=Normal&wdorigin=ItemsView&wdhostclicktime=1779371568742&afdflight=27&csiro=1&instantedit=1&wopicomplete=1&wdredirectionreason=Unified_SingleFlush#sec-specify)). 2. Cite this checklist (See [Note 2](https://auc-word-edit.officeapps.live.com/we/wordeditorframe.aspx?ui=en-GB&rs=en-AU&wopisrc=https%3A%2F%2Fcharlesdarwinuni.sharepoint.com%2Fteams%2FClinicalTrials%2F_vti_bin%2Fwopi.ashx%2Ffiles%2Ff10e2025357e4157ab3983f16d92bb11&wdenableroaming=1&wdfr=1&mscc=1&hid=C4CF15A2-80A9-7000-82FB-75B1748C5A36.0&uih=sharepointcom&wdlcid=en-GB&jsapi=1&jsapiver=v2&corrid=59a17e2b-b656-7e7f-4a3d-3bebec4f37d9&usid=59a17e2b-b656-7e7f-4a3d-3bebec4f37d9&newsession=1&sftc=1&uihit=docaspx&muv=1&ats=PairwiseBroker&cac=1&sams=1&mtf=1&sfp=1&sdp=1&hch=1&hwfh=1&dchat=1&sc=%7B%22pmo%22%3A%22https%3A%2F%2Fcharlesdarwinuni.sharepoint.com%22%2C%22pmshare%22%3Atrue%7D&ctp=LeastProtected&rct=Normal&wdorigin=ItemsView&wdhostclicktime=1779371568742&afdflight=27&csiro=1&instantedit=1&wopicomplete=1&wdredirectionreason=Unified_SingleFlush#sec-cite)). 3. Include your completed checklist as a supplement when submitting to a journal so that future readers can use it to find information. |

|  | Item Description | Location (or reason for not reporting) |
| --- | --- | --- |
| **Title & Abstract** |  |  |
| [Title](https://resources.equator-network.org/reporting-guidelines/srqr/items/title.html) | Describe the nature and topic of the study. Identify the study as qualitative or indicate the approach or data collection methods. | Title page |
| [Abstract](https://resources.equator-network.org/reporting-guidelines/srqr/items/abstract.html) | Summarise the key elements of the study using the abstract format of the intended publication. | Pgs. 2-3; Abstract |
| **Introduction** |  |  |
| [Problem Formulation](https://resources.equator-network.org/reporting-guidelines/srqr/items/problem-formulation.html) | Describe the problem/phenomenon studied, its significance, relevant theory and empirical work, and gaps in current knowledge. | Pgs. 5-7; Background; paragraphs 1-6; |
| [Purpose or research question](https://resources.equator-network.org/reporting-guidelines/srqr/items/purpose.html) | Describe the purpose of the study and specific objectives or questions. | Pg. 8; Methods; Study Aim and Design; para 1 |
| **Methods** |  |  |
| [Qualitative approach and research paradigm](https://resources.equator-network.org/reporting-guidelines/srqr/items/qualitative-approach.html) | Describe your qualitative approach, your guiding theory (if appropriate), and research paradigm, and reasons for your choices. | Pg. 6; Background; para 6    Pg. 8; Methods; Study Aim and Design; para 1 |
| [Researcher characteristics and reflexivity](https://resources.equator-network.org/reporting-guidelines/srqr/items/researcher-characteristics-and-reflexivity.html) | Describe how researchers’ characteristics may influence the research, including personal attributes, qualifications/experience, relationship with participants, assumptions, and/or presuppositions; potential or actual interaction between researchers’ characteristics and the research questions, approach, methods, results and/or transferability. | Pg. 11: Reflexivity;    Supplementary File 3 |
| [Context](https://resources.equator-network.org/reporting-guidelines/srqr/items/context.html) | Describe the setting/site(s) in which the study was conducted, why it was selected, and any other salient contextual factors that may influence the study. | Pg. 8-9; Methods; Study Setting and Context; paragraphs 1 & 2 |
| [Sampling strategy](https://resources.equator-network.org/reporting-guidelines/srqr/items/sampling-strategy.html) | Describe how and why research participants, documents, or events were selected; criteria for deciding when no further sampling was necessary, and the rationale for those criteria. | Pg. 9-10; Methods; Participants; para 1 |
| [Ethical issues pertaining to human subjects](https://resources.equator-network.org/reporting-guidelines/srqr/items/ethics.html) | Describe any approval by an appropriate ethics review board and participant consent, or explain any lack thereof. Describe any other confidentiality and data security issues. | Pg. 45; Declarations: paragraphs 1-3    Pg. 10; Methods; Data Collection; para 2 |
| [Data collection methods](https://resources.equator-network.org/reporting-guidelines/srqr/items/data-collection-methods.html) | Describe the types of data collected; details of data collection procedures including (as appropriate) start and stop dates of data collection and analysis, iterative process, triangulation of sources/methods, and modification of procedures in response to evolving study findings. Describe your rationale for these choices. | Pg. 10; Methods; Data Collection; paragraphs 1 & 2 |
| [Data collection instruments and technologies](https://resources.equator-network.org/reporting-guidelines/srqr/items/data-collection-instruments.html) | Describe any instruments (e.g., interview guides, questionnaires) and devices (e.g., audio recorders) used for data collection; describe if/how the instrument(s) changed over the course of the study. | Pg. 10; Methods; Data Collection; paragraphs 1 & 2 |
| [Units of study](https://resources.equator-network.org/reporting-guidelines/srqr/items/units-of-study.html) | Describe the number and relevant characteristics of participants, documents, or events included in the study. Describe the level of participation. | Pg. 13; Results; Participant Characteristics |
| [Data processing](https://resources.equator-network.org/reporting-guidelines/srqr/items/data-processing.html) | Describe the methods for processing data prior to and during analysis, including transcription, data entry, data management and security, verification of data integrity, data coding, and anonymisation / deidentification of excerpts. | Pg. 10; Methods; Data Collection; para 2    Pg. 11; Methods; Data Analysis |
| [Data analysis](https://resources.equator-network.org/reporting-guidelines/srqr/items/data-analysis.html) | Describe the process by which inferences, themes, etc. were identified and developed, including the researchers involved in data analysis; usually references a specific paradigm or approach. Describe why you chose this process. | Pg. 11; Methods; Data Analysis |
| [Techniques to enhance trustworthiness](https://resources.equator-network.org/reporting-guidelines/srqr/items/trustworthiness.html) | Describe any techniques to enhance trustworthiness and credibility of data analysis,(e.g., member checking, triangulation, audit trail). Describe why you chose these techniques. | Pg. 11; Methods; Data Analysis |
| **Results** |  |  |
| [Synthesis and interpretation](https://resources.equator-network.org/reporting-guidelines/srqr/items/synthesis-and-interpretation.html) | Describe the main findings (e.g., interpretations, inferences, and themes); might include development of a theory or model, or integration with prior research or theory. | Pgs. 13-25; Results |
| [Links to empirical data](https://resources.equator-network.org/reporting-guidelines/srqr/items/links-to-empirical-data.html) | Provide evidence (e.g., quotes, field notes, text excerpts, photographs) to substantiate analytic findings. | Pgs. 13-25; Results |
| **Discussion** |  |  |
| [Integration with prior work, implications, transferability, and contribution(s) to the field](https://resources.equator-network.org/reporting-guidelines/srqr/items/integration-with-prior-work.html) | Summarize the main findings, explain how findings and conclusions connect to, support, elaborate on, or challenge conclusions of earlier scholarship; discuss the scope of application/generalizability; identify unique contribution(s) to scholarship in a discipline or field. | Pgs. 26-32; Discussion |
| [Limitations](https://resources.equator-network.org/reporting-guidelines/srqr/items/limitations.html) | Discuss the trustworthiness and limitations of findings | Pg. 33; Limitations |
| **Other** |  |  |
| [Conflicts of interest](https://resources.equator-network.org/reporting-guidelines/srqr/items/conflicts-of-interest.html) | Describe any potential sources of influence or perceived influence on study conduct and conclusions. Describe how these were managed. | Pg. 45; Declarations; Competing interests |
| [Funding](https://resources.equator-network.org/reporting-guidelines/srqr/items/funding.html) | Describe sources of funding and other support. Describe the role of funders in data collection, interpretation, and reporting. | Pg. 45; Declarations; Funding |

O’Brien BC, Harris IB, Beckman TJ, Reed DA, Cook DA. The SRQR reporting checklist. In: Harwood J, Albury C, Beyer J de, Schlüssel M, Collins G, editors. The EQUATOR network reporting guideline platform [Internet]. The UK EQUATOR Centre; 2025. Available from: https:/resources.equator-network.org/reporting-guidelines/srqr/srqr-checklist.docx

1. FMOH. National Human Resource for Health Strategic Plan for Ethiopia 2016-2025. Addis Ababa; 2016 Sep. [↑](#footnote-ref-1)
2. Feysia B, Herbst CH, Lemma W, Soucat A. The Health Workforce in Ethiopia: Addressing the Remaining Challenges. Washington; 2012. [↑](#footnote-ref-2)
